# Food Additives Associated with Gut Microbiota Alterations in Inflammatory Bowel Disease: Friends or Enemies?

**DOI:** 10.3390/nu14153049

**Published:** 2022-07-25

**Authors:** Caiguang Liu, Shukai Zhan, Zhenyi Tian, Na Li, Tong Li, Dongxuan Wu, Zhirong Zeng, Xiaojun Zhuang

**Affiliations:** 1 Department of Gastroenterology, The First Affiliated Hospital, Sun Yat-sen University, Guangzhou 510080, China; liucg3@mail2.sysu.edu.cn (C.L.); zhanshk@mail2.sysu.edu.cn (S.Z.); lina87@mail2.sysu.edu.cn (N.L.); litong68@mail.sysu.edu.cn (T.L.); wudx6@mail2.sysu.edu.cn (D.W.); 2 Department of Gastroenterology, Zhujiang Hospital, Southern Medical University, Guangzhou 510282, China; tianzhy3@mail2.sysu.edu.cn

**Keywords:** food additive, gut microbiota, inflammatory bowel disease

## Abstract

During the 21st century, the incidence and prevalence of inflammatory bowel disease (IBD) is rising globally. Despite the pathogenesis of IBD remaining largely unclear, the interactions between environmental exposure, host genetics and immune response contribute to the occurrence and development of this disease. Growing evidence implicates that food additives might be closely related to IBD, but the involved molecular mechanisms are still poorly understood. Food additives may be categorized as distinct types in accordance with their function and property, including artificial sweeteners, preservatives, food colorant, emulsifiers, stabilizers, thickeners and so on. Various kinds of food additives play a role in modifying the interaction between gut microbiota and intestinal inflammation. Therefore, this review comprehensively synthesizes the current evidence on the interplay between different food additives and gut microbiome alterations, and further elucidates the potential mechanisms of food additives–associated microbiota changes involved in IBD.

## 1. Introduction

Inflammatory bowel diseases (IBD), which included Crohn’s disease (CD) and ulcerative colitis, are featured as chronically recurrent inflammatory disorders of the digestive tract. In recent decades, IBD has affected about 150–200 cases per 100,000 in western countries. Moreover, its prevalence in many newly industrialized countries presents a trend that increases fleetly, indicating the critical player of environmental factors as the disease progresses [1]. To date, the etiology of IBD is still uncertain. The interactions between environmental triggers and gut microbiome alterations in genetically susceptible individuals could stimulate an aberrant immune disorder and drive chronic gut inflammation. As an important environmental factor, dietary patterns may have the pivotal role in altering intestinal floras, followed by dysregulated host homeostasis and immunological processes [2].

Many investigations have focused on the contributing impact of different dietary patterns or eating habits on IBD [3]. The western diet, a kind of food pattern with an increased consumption of total fat, unsaturated fatty acids, refined proteins and processed carbohydrates, was correlated with a higher risk of IBD flare-ups. Mechanistically, an excessive intake of saturated fatty acids, sugars and processed meats could induce intestinal barrier dysfunction and low-grade intestinal inflammation [4]. Diet-induced changes in the intestinal microbiota composition involved in IBD also appear [5]. Furthermore, the western diet might be accompanied by an increased consumption of food additives. Food additives are defined as chemical synthesis or natural substances improving food quality, prolonging the food storage period, facilitating the food process and enriching food nutrients [6]. The appliance of food additives must follow the guidelines and relevant regulations authorized by the Food and Drug Administration (FDA) in the United States or the European Food Safety Authority (EFSA) in Europe. On the basis of their function and property, food additives can be categorized into different groups such as preservatives, artificial sweeteners, food colorants, flavor enhancers, emulsifiers, stabilizers and thickeners, anti-caking agents and so on [7]. Nevertheless, recent studies pointed out that some of the approved food additives might exert deleterious effects [8]. Food additives permitted in the European Union before 20 January 2009 had to be re-evaluated by 31 December 2020. More seriously, the underlying mechanisms of various food additives involved in IBD are still uncertain.

Clinical and experimental data suggested that gut dysbiosis and gut microbiota-derived metabolites are closely related to the onset and progression of IBD. The diversity of gut microbiota in IBD patients was reduced, with decreased short-chain fatty acids (SCFAs) producing bacteria as well as increased mucolytic and pathogenic bacteria [9]. Several metabolites including bile acid derivatives, SCFAs and tryptophan metabolites have been highlighted [9]. Moreover, the gut microbiota was hypothesized as a medium, linking food additive intake to the intestinal inflammation of IBD. For instance, dietary emulsifiers were supposed to induce low-grade inflammation via microbiota disturbance [10]. These emulsifiers also decreased the level of SCFAs and altered mucus thickness to aggravate intestinal inflammation in interleukin-10 deficient (IL10 KO) and toll-like receptor 5 knockout mice [11]. Interestingly, gut microbiota alterations caused by food additives are somehow in accordance with those in IBD patients. Hence, the relation between food additives and intestinal microbiota might help to better interpret the occurrence and development of IBD.

This review synthesizes the existing research concerning food additive-driven gut microbiota alterations involved in intestinal inflammation and further elucidates the possible mechanisms of food additives involved in IBD.

## 2. Artificial Sweeteners 

Artificial sweeteners, called non-caloric artificial sweeteners (NAS), are sugar substitutes without adding calories or triggering a blood glucose response. In accordance with Article (5) of the Regulation (EU) No. 257/2010, EFSA has already made open calls for data on sweeteners under the re-evaluation program. On the basis of the information received from interested parties and those retrieved from the literature, the assessment of these food additives has been started [12,13]. Recently, a call for data on genotoxicity data on sweeteners was also proclaimed by EFSA. Artificial sweetener-induced gut microbiota and metabolites alterations have been observed in current research, such as aspartame, acesulfame-K (Ace-K), stevia, sucralose, saccharin, neotame and the corresponding compounds. Details were shown in Table 1, Table 2 and Supplementary Table S1.

### 2.1. Aspartame (E 951)

Aspartame (E 951) is a low-calorie and intense artificial sweetener. In Europe, it is authorized for use as a food additive in various foodstuffs and as a table-top sweetener. EFSA published its first full risk assessment of aspartame in December 2013 and the opinion concluded that aspartame and its breakdown products are safe for general population (including infants, children and pregnant women) [14]. However, people with phenylketonuria have a difficult time metabolizing phenylalanine and should control the intake of aspartame. In humans, aspartame might not alter the abundance but the diversity of fecal microbiota [15,16]. However, the increases in SCFAs-producing bacteria such as *Bifidobacterium* and *Blautia coccoides* and the decreases in the ratio of *Bacteroides/Prevotella* were detected in another study [17]. In animal experiments, the abundance of *Firmicutes* and *Clostridium leptum* was higher and the richness of *Enterococcus* and *Parasutterella* was lower after aspartame treatment [18,19]. The concentrations of SCFAs, including propionic and butyric acid, were also increased in serum, feces and the cecal contents (Table 2) [18,19]. In vitro treatment by NAS mixtures (aspartame and Ace-K) stimulated *Escherichia coli* (*E. coli*) expansion. The enoyl ACP reductase, a rate-limiting enzyme for the butyrate biosynthesis, was also overexpressed [20]. Although few studies explored the relationships between aspartame, gut microbiota and IBD, this substance might be a “friendly” food additive in IBD by enriching SCFAs-producing bacteria and the concentration of SCFAs.

### 2.2. Acesulfame K (E 950)

Ace-K, or acesulfame potassium, is a commonly used NAS. The acceptable daily intake (ADI) of Ace-K was 9 mg/kg body weight (bw), which is also suitable for children 1–3 years old for special medical purposes and is considered not a safety concern [21]. In animal experiments, the amounts of total bacteria in an Ace-K treatment group were equivalent to the control group [22]. However, in another study, Ace-K consumption showed as highly gender-specific in changing gut microbiota and metabolites. In females, the lower abundance of *Lactobacillus* and *Clostridium* genera and the higher level of *Mucispirillum* genus were observed, while in males, the abundances of *Bacteroides*, *Anaerostipes* and *Sutterella* were increased [23]. Exposing mice to sucralose and Ace-K during pregnancy and lactation might change the α- and β-diversity of their offspring’s gut microbiota, showing an increase in *Firmicutes* and an extreme decrease in the potential anti-inflammatory bacteria *Akkermansia muciniphil* [24]. Ace-K administration after antibiotic treatment also induced an expansion of the sulfate-reducing bacteria *Desulfovibrio* and a higher expression of proinflammatory cytokines in the colon. Moreover, Ace-K might evoke indomethacin-induced intestinal damage via dysbiosis (shown in Table 1) [25]. However, Ace-K would inhibit the growth of *E. coli* [26], which is contrary to the finding from Mahmud et al., [20]. 

In the fecal samples of IBD patients, there was a high level of primary bile acids such as cholic acid (CA) and a low concentration of secondary bile acids such as deoxycholic acid (DCA) [27]. Interestingly, the change trends of CA and DCA were consistent in mice treated with Ace-K [22]. Some genes related to lipopolysaccharide (LPS) and flagella synthesis were also upregulated (Table 2) [23]. To sum up, a decrease of anti-inflammatory bacteria with some bacterial function alterations such as LPS and bile acid synthesis were reported after treatment with Ace-K. This suggests that gut microbiome and metabolite perturbation induced by Ace-K might be the key disruptor for intestinal homeostasis, which might be an increased risk for IBD.

**Table 1:** The interactions among food additives, gut microbiota and inflammatory bowel disease.

Food Additives	Study	Model	Sample and Gut Microbiota Alterations	Inflammatory Effects
Artificial sweeteners				
Ace-K	Hanawa et al., 2019 [25]	Mice	(F) Increase: *Desulfovibrio* in genus.	Induced the expression of inflammatory cytokines.
Sucralose	Wang et al., 2018 [8]	Rats with TNBS-induced colitis	(F) Increase: *Proteobacteria* and *Bacteroidetes;* Decrease: *Firmicutes* and *Actinomycetes.*	Exacerbated colitis, aggravated changes in colon length, MPO, TNF-α and IL-1β in gut tissue.
Sucralose	Li et al., 2020 [28]	Mice with DSS/AOM-induced colon cancer	(F) Increase: *F. Actinomycetes*, *P. stomatis*, *C. symbiosum*, *P. anaerobius* Decrease: *Proteobacteria*	Aggravation of colorectal tumors; induction of inflammatory cytokines and pathways (TNF-α, IL-1β, IL-6, IL-10).
Splenda	Rodriguez-Palacios et al., 2018 [29]	Ileitis-prone SAMP mice	(F) Increase: *Proteobacteria* and *E. coli* with increased bacterial infiltration into the lamina propria; malX gene–carrying bacterial	Increase MPO activity; no impact on the severity of ileitis.
Saccharin	Sünderhauf et al, 2020 [30]	Mice with DSS-induced colitis	(F) Influenced on β-diversity Increase: *Bacteroidetes* and *Proteobacteria* phylum Decrease: *S. aureus*, *K. pneumonia* and *P. aeruginosa*	Improved intestinal inflammation with less weight loss, lower DAI and histological score.
Sugar alcohols				
Lactitol	Wang et al., 2019 [31]	Mice with DSS-induced colitis	(F) Altered the α-diversity; increase: *Akkermansia*	Improved inflammation in acute colitis mice.
Coating and thickening agents				
MDX	Zangara et al., 2021 [32]	IL10 KO and NOD2 KO mice	(F) Decrease in α-diversity; altered β-diversity	Accelerated the onset of colitis; elevated intestinal infiltration of CD3+ cells and intestinal pathology; reduced mucin granule content.
MDX	Thymann et al., 2009 [33]	Pigs with NEC	(IC) Lower the bacterial diversity Increase: *Pseudomonas* spp., *Streptococcus* spp., *Leuconostoc* spp. Decrease: *Weissella app*	Induced higher incidence of NEC; reduced villus height.
MDX	Kourtney et al., 2009 [34]	Mice with *Salmonella* gastroenteritis	Enhances mucosal *Salmonella* colonization in vivo	Wrecked the intestinal antimicrobial barrier in vivo. Suppressed NAPDH oxidase expression; reduced recruitment of NADPH oxidase to *Salmonella*-containing vesicles, resulting in persistence of *Salmonella* in vesicles.
MDX	Kourtney et al., 2009 [35]	AIEC isolated from patients	MDX enhanced AIEC specific biofilm formation	Induced type I pili expression; increased bacterial adhesion to intestinal epithelial.
Emulsifiers				
P80	Hirotaka et al., 2019 [36]	Mice with indomethacin-induced colitis	(IC) Decreased the α-diversity in the small intestine Increased: *Gammaproteobacteria* and *P. mirabilis*	Exacerbated colitis; increased the interleukin-1β expression. Antibiotic pretreatment abolished this effect.
P80	Roberts et al., 2010 [37]	*E coli* isolates from patients	-	Increased the translocation of *E coli* across M epithelial cells.
CMC	Zangara et al., 2021 [32]	IL10 KO mice and NOD2 KO mice	(F) Flagella expression by microbes was elevated	Accelerated the onset of colitis; elevated intestinal infiltration of CD3+ cells and intestinal pathology; reduced mucin granule content.
CMC	Swidsinski et al., 2011 [38]	IL10 KO mice	(Intestinal mucosa) Bacterial overgrowth	Distention of spaces between villi, with bacteria filling these spaces, adherence of bacteria to the mucosa and migration of bacteria to the bottom of the crypts.
CMC and P80	Chassaing et al., 2017 [39]	M-SHIME; ASF and GF mice	In vitro: influenced on diversity and composition (F) Increase in inflammation-related bacteria, decreased health-associated bacteria	Promoted low-grade gut inflammation.
CMC and P80	Chassaing et al., 2015 [11]	Wildtype, IL10 KO and TLR5 KO mice;	(F) Induced a reduction in microbial diversity Increase: *Verrucomicrobia* phylum, *A. muciniphila*, *Proteobacteria*	Induced low-grade intestinal inflammation and promoted robust colitis.
CMC and P80	Viennois et al., 2020 [10]	IL10 KO and ASF/GF mice; DSS-induced colitis	-	Induced chronic intestinal inflammation and metabolism dysregulations, especially in IL10 KO.
Carrageenan	Li et al., 2014 [40]	GF mice	GF mice inoculated with *B. xylanisolvens 38F6A4 or E. coli 38F6C1*	Increased intestinal permeability and was related to the onset of colitis.
Carrageenan	Shang et al., 2017 [41]	Mice	(CC) Decrease: *A.muciniphila*	Induced low-grade colitis.
Carrageenan	Ye et al., 2020 [42]	Mice with HFD induced-colitis	(F) Increase: *A. finegoldii* and *B. acidifaciens*	Aggravated intestinal inflammation in colitis mice.
Carrageenan	Wu et al., 2017 [43]	Mice with *Citrobacter freundii* DBS100-induced colitis	-	Aggravated intestinal inflammation in colitis mice.
Carrageenan	End et al., 2009 [44]	Mice with DSS-induced colitis	Inhibits the bacterial aggregating function of DMBT1	Disrupts the mucosal protection provided by DMBT1.
Carrageenan	Munyaka et al., 2016 [45]	Mice inoculated with AIEC	(IC) Decreased bacterial richness and composition Increase: *Proteobacteria* and *Deferribacteres* Decrease: *Firmicutes*, *Actinobacteria, Bacteroidetes*	Induced colitis in mice.
Carrageenan	Onderdonk et al., 1978 [46]	Guinea pigs	-	Induced the cecal ulcerations; no effect on GF pigs.
Carrageenan	Onderdonk et al., 1983 [47]	Guinea pigs; GF mice	-	Inoculated with *B. vulgatus* developed cecal ulcerations.
GML	Mo et al., 2019 [48]	Mice	(F) Increase: *Barnesiella*; *Clostridium XIVa*, *Oscillibacter*, *Parasutterella*	Maintained intestine barrier; promoted anti-inflammatory environment.
GML	Zhao et al., 2020 [49]	Mice with HFD	(F) Increase: *Bifidobacterium pseudolongum*	Ameliorated the metabolic disorders and gut inflammation.
GML	Zhao et al., 2019 [50]	Mice with HFD	(F) GML ameliorates gut microbiota dysbiosis Increase: *B. uniformis*, *Akkermansia*, *Bifidobacterium*, *Lactobacillus* Decrease: *E. coli*, *Lactococcus*, *Flexispira*	Ameliorates metabolic disorders and reduced serum TNF-α.
GML	Mo et al., 2021 [51]	Mice with DSS-induced colitis	(F) Increase: *Lactobacillus* and *Bifidobacterium* Decrease: *Helicobacter ganmani*	Improved colitis in mice.
Food colorants				
TiO_2_	Cao et al., 2020 [52]	Mice with HFD	(F)Increase: *Firmicutes* Decrease: *Bacteroidetes*, *Bifidobacterium*, *Lactobacillus*	Induced strong colonic inflammation, especially in obese mice.
TiO_2_	Zhu et al., 2021 [53]	Mice with HFD	(F) Increase: *Firmicutes*; Decrease: *Bacteroidete*	Escalated the low-grade inflammation induced by HFD through gut microbiome; disrupted mucus layer.
TiO_2_	Yan et al., 2020 [54]	Mice	(CC) Decrease: *Akkermansia, Barnesiella*, *Bacteroides* Increase: *Barnesiella*	Caused intestinal inflammation; reduced intestinal mucus barrier.
TiO_2_	Kurtz et al., 2020 [55]	Mice	(CC) Affected the colonization of mucosa-associated bacteria	Elicits an inflammatory response in ileum.
TiO_2_	Chen et al., 2019 [56]	Rats	(F) Increase: *L. gasseri*, *Turicibacter*, *L. NK4A136* group Decrease: *Veillonella*	Induced inflammatory infiltration and mitochondrial abnormalities.
TiO_2_	Pinget et al., 2019 [57]	Mice	(F) Promoted biofilm formation by *E. faecalis* or *E. coli*	Wrecked the gut barrier and induced gut inflammation.
TiO_2_	Mu et al., 2019 [58]	Mice with DSS-induced colitis	(F) Affected the diversity Decrease: *Bifidobacterium*, *Lactobacillus*	Induced intestinal inflammation; aggravated colitis.
TiO_2_	Chen et al., 2017 [59]	Mice with DSS-induced colitis	(F) No influence	No influence.
Azo dyes	He et al., 2021 [60]	GF, Rag1-/-and R23FR mice	(F) No influence on bacterial composition.	Red 40 and ANSA-Na promoted colitis.
Azo dyes	Wu et al., 2021 [61]	Crucian carp	(IC) Increase: *Bdellovibrio Shewanella* Decrease: *Roseomonas*, *Rhodococcu*, *Bacillus*, *Bacteroides*, *Clostridium*	Induced the oxidative stress; elicited a tendency to gut inflammation.
Food preservatives				
Mixture	Hrncirova et al., 2019 [62]	Wildtype, NOD2 KO mice	(F) Increase: *Proteobacteria* phylum Decrease: *Clostridiales* order	Dysbiosis was induced, especially in the NOD2 KO mice.
Sulfite	Schooth et al., 2020 [63]	*P. mirabilis*, *M. morganii*, *E. fergusonii*, *K. pneumoniae*	Reduced the growth rate of all strains.	Influenced the growth kinetics of Crohn’s disease pathobionts, which may initiate and promote disease.
TCS	Yang et al., 2018 [64]	Mice with DSS-in duced colitis; IL10 KO mice	(F) Lower the α- and β-diversity Increase: *Firmicutes* Decrease: *Bacteroidetes*, *Actinomycetes*, *Cyanobacteria*	Induced low-grade colonic inflammation, increased colitis, and exacerbated colitis-associated colon cancer in mice

Abbreviations: Ace-K—acesulfame K; MDX—maltodextrin; CMC—carboxymethylcellulose; P80—polysorbate 80; GML—Glycerol monolaurate; TiO_2_—titanium dioxide nanoparticles; Mixture—a mixture of common preservatives including benzoate, nitrite and sorbate; TCS—Triclosan; TNBS—2, 4, 6, trinitrobenzene sulphonic acid; DSS—dextran sulfate sodium; IL10 KO mice—interleukin-10 deficient mice; NOD2 KO mice—nucleotide-binding oligomerization domain 2 deficient mice; TLR5 KO mice—toll-like receptor 5 knockout mice; NEC—necrotizing enterocolitis; SAMP—SAMP1/YitFc; AIEC—Crohn’s disease-associated adherent invasive *E. coli*; ASF mice—altered Schaedler flora mice; GF mice—germ free mice; M-SHIME—the mucosal simulator of the human intestinal microbial ecosystem model; HFD—high-fat diet; F—feces; IC—the intestinal content; CC—the colon content; T, MPO—myeloperoxidase; DAI—disease activity index; ANSA-Na—1-amino-2-naphthol-6-sulfonate sodium salt.

**Table 2:** The influence of various food additives on the intestinal metabolites.

Food Additives		Study	Sample	Metabolite Alterations	
				Increase	Decrease
Artificial sweeteners	Aspartame	Gerasimidis et al., 2021 [17]	F	Total SCFAs, acetic acid, propionic acid, caprylic acid	Valeric acid, caproic acid; BCFAs (such as isobutyric acid, isovaleric acid)
		Palmnäs et al., 2014 [18]	S	Propionate, acetate and butyrate	-
		Jodi et al., 2020 [19]	CC	Propionate, butyrate and isobutyrate	-
	Sucralose	Uebanso et al., 2017 [22]	CC	The CA/CDCA ratio	-
		V amanu et al., 2019 [65]	F	Ammonium, formic acid, phenyllactic acid, HO-phenyllactic acid; butyric acid	Benzoic acid
	Saccharin	V amanu et al., 2019 [65]	F	Ammonium, formic acid, phenyllactic acid, HO-phenyllactic acid; acetic and butyric acid	Benzoic acid, propionic acid
		Suez et al., 2014 [66]	F	Propionate and acetate	-
		Bian et al., 2017 [67]	F	Daidzein, dihydrodaidzein and O-desmethylangolensin; quinolinic acid	Equol, linoleoyl, ethanolamide, N, *N*-Dimethylsphingosine
	Neotame	Liang et al., 2018 [68]	F	Cholesterol, campesterol and stigmastanol	Malic acid, mannose-6-phosphate, 5-aminovaleric acid and glyceric acid; 1, 3-dipalmitate, 1-monopalmitin, linoleic acid and stearic acid
	Cyclamate	V amanu et al., 2019 [65]	F	Formic aid, phenyllactic acid, HO-phenyllactic acid; acetic acid	Benzoic acid, propionic acid
	Splenda	Karley et al., 2019 [69]	F	Butyric and pentanoic acid	-
Sugar alcohols	Isomalt	Gostner et al., 2016 [70]	F	No influence on SCFAs, lactate, bile acids and neutral sterols.	
	Lactitol	Chu et al., 2019 [71]	F	No influence on SCFAs.	
		Ballongue et al., 2016 [72]	F	Acetic acid, lactic acids	Propionic, butyric and valeric acids
		Finney et al., 2007 [73]	F	Propionic and butyric acids	Acetic acid, lactic acids
		Peuranen et al., 2004 [74]	F	Butyrate	-
		Pinna et al., 2014 [75]	IC	Putrescine	The acetic acid to propionic acid ratio
Coating and thickening agents	MDX	Gerasimidis et al., 2020 [17]	F	Total SCFAs, propionic acid; caprylic acid	Valeric acid, caproic acid; isobutyric and isovaleric acid
		Thymann et al., 2009 [33]	IC	Formic acid, acetic acid, butyric acid	Lactic acid, succinic acid
		Kong et al., 2020 [76]	F	Total SCFAs, acetate, butyrate and valerate	-
Emulsifiers	P80	Chassaing et al., 2015 [11]	F	Flagellin	-
	CMC	Chassaing et al., 2017 [39]	F	Butyrate; LCA, HDCA/UDCA, αMCA, GLCA, TCDCA, TDCA, THDCA/TUDCA, TCA	-
		Chassaing et al., 2015 [11]	F	Butyrate, heptanoate; αMCA	-
		Gerasimidis et al., 2020 [17]	F	-	Isovaleric acid
	Carrageenan	Gerasimidis et al., 2020 [17]	F	No influence on SCFAs or BCFAs.	
		Munyaka et al., 2016 [45]	CC	-	Butyric and acetic acid
Food colorants	TiO_2_	Cao et al., 2020 [52]	CC	-	Butyric and propionic acid; acetic and isovaleric acids in obese mice
		Chen et al., 2019 [56]	F	*N*-acetylhistamine, caprolactam and glycerophosphocholine	4-methyl-5-thiazoleethanol, L-histidine and L-ornithine
		Pinget et al., 2019 [57]	S	-	SCFAs
		Waller et al., 2017 [77]	CC	-	pH level
		Agans et al., 2019 [78]	F	No influence on SCFAs.	
		Dudefoi et al., 2017 [79]	F	No influence on overall fatty acid compositions.	
		Gerasimidis et al., 2021 [17]	M	No influence on SCFAs or BCFAs.	
	Azo dyes	Polic et al., 2018 [80]	M	-	Acetate, butyrate and propionate
		Chen et al., 2009 [81]	M	Metabolites of Sudan III and IV, aniline and o-toluidine (2-methylaniline) were carcinogenic aromatic amines	
		Pan el al, 2012 [82]	M	1-Amino-2-naphthol, a common metabolite of the dyes, was capable of inhibiting growth of most of the tested bacteria	
Preservatives	Benzoic acid	Torrallardona et al., 2007 [83]	U	Hippuric acid	-
		Kluge et al., 2005 [84]	IC	-	Acetic acid
		Diao et al., 2013 [85]	CC	Propionic acid and total volatile fatty acid	NH3–N
		Diao et al., 2014 [86]	CC	Butyric acid	-
	Ag NPs	Cueva et al., 2019 [87]	F	Ammonium	-
Antioxidant	Rosemary extract	Romo-Vaquero et al., 2014 [88]	F	SCFAs (acetic, propionic and butyric acid) in obese mice	SCFAs in lean mice

Abbreviations: MDX—maltodextrin; CMC—carboxymethylcellulose; P80—polysorbate 80; TiO_2_—titanium dioxide nanoparticles; Ag NPs—Ag nanoparticles; F—feces; S—serum; CC—the colon content; IC—the intestinal content; U— urine; M—culture medium in vitro study; SCFAs—short-chain fatty acids; BCFAs—branched-chain fatty acids; CD—cholic acid; CDCA—chenodeoxycholic acid; LCA—lithocholic acid; HDCA—hyodeoxycholic acid; UDCA—ursodeoxycholic acid; αMCA—α-muricholic acid; GLCA—glycolithocholate; TCDCA—taurochenodeoxycholic acid; TDCA—taurodeoxycholic acid; THDCA—taurohyodeoxycholic acid; TUDCA—tauroursodeoxycholic acid; TCA—taurocholic acid.

### 2.3. Sucralose (E 950)

Sucralose, known as trichlorogalactosucrose, has a 600–650 times higher sweetness than sugar. In human studies, the intake of sucralose did not modulate the gut microbiome in a short treatment [16,89], while a ten-week consumption in young adults might lead to an increase in *Blautia coccoides* and a decrease in *Lactobacillus acidophilus*, with altered insulin and glucose levels in the serum [90]. However, Uebanso et al., showed the decreased amount of *Clostridium IVXa* in feces with a dose-dependent manner in animals [22]. A total of 14 genera were strikingly changed after 3 or 6 months of treatment (i.e., *Ruminococcus* increased at 3 months; *Turicibacter*, *Roseburia* and *Akkermansia* increased at 6 months; *Anaerostipes*, *Staphylococcus* and *Bacillales* decreased at 3 months; *Streptococcus* decreased at 6 months) [91]. Similar results were found in high-fat diet-fed mice models [26]. Splenda is an NAS and mainly consists of sucralose and maltodextrin. It was supposed to inhibit the growth of total anaerobes including *Bifidobacteria*, *Lactobacilli*, *Bacteroides* and *Clostridia* [8,29]. V amanu et al., investigated the alteration of human intestinal floras through a vitro static system called *GIS1* and revealed similar results, with decreases in the beneficial bacteria such as *Bifidobacterium* genus and increases in the possibly pathogenic bacteria *Enterococcaceae* genus [65]. The growth of bile-tolerant microorganisms *Bilophila* genus, the anti-inflammatory bacteria *Faecalibacterium prausnitzii* and two species from the *Bacteroides* genus (*Bacteroides fragilis* and *Bacteroides uniformis*) were inhibited after culturing with sucralose in vitro [17,26,92,93].

The relationship among sucralose, gut microbiota and intestinal inflammation were directly investigated in three studies and demonstrated in Table 1. In one study, SAMP1/YitFc mice administered with Splenda did not aggravate colitis but increased the level of myeloperoxidase (MPO) in colon tissue. The fecal microbiota analysis found that *Proteobacteria* phylum was elevated, but the levels of *Lactobacilli* and *Clostridia* were decreased [29]. On the contrary, Splenda could increase the susceptibility of 2, 4, 6, trinitrobenzene sulphonic acid-induced colitis, with elevated levels of *Bacteroidetes* and *Proteobacteria* and reduced amounts of *Firmicutes* and *Actinomycetes* [8]. A recent investigation also found that sucralose could promote the risk of colitis-associated colorectal cancer. The abundance of *Actinomycetes* and the three species *Peptostreptococcus stomatis*, *Clostridium symbiosum* and *Peptostreptococcus anaerobius* were increased, but that of *Proteobacteria* was reduced [28].

The consumption of sucralose resulted in multiple metabolites alterations (Table 2). The level of tyrosine was increased, while *p*-hydroxyphenylacetic acid and cinnamic acid were decreased. These compounds can restrain the production of reactive oxygen species (ROS), which are involved in tryptophan metabolism. The content of bile acids was impaired, with a greater concentration of CA and a higher CA/chenodeoxycholic acid ratio in the cecal content. The researchers suggested that these metabolite changes would result in triggering and maintaining liver inflammation [22,91]. On the basis of existing evidence, we supposed that sucralose is detrimental to colitis with a potential to increase the abundance of proinflammatory bacteria. Nevertheless, the actual alternations in gut microbiota and metabolites caused by sucralose remained uncertain and suggests a need for further clinical investigations.

### 2.4. Saccharin (E 954)

Most saccharin will be absorbed and finally eliminated by urine, while the non-absorbed saccharin would be excreted by feces. Although a recent study found that a high-dose supplementation does not induce gut microbiota changes or glucose intolerance [94], saccharin consumption led to an enrichment of mucosal inflammatory cells and changed the gut permeability in mice. In addition, saccharin-exposed mice pretreated with antibiotics induced a lower level of mucosal inflammation and gut barrier dysfunction [95]. These findings indicated that gut dysbiosis is considered to mediate these abnormalities.

The high level of saccharin in the cecal contents was associated with an increase in the aerobic population [96]. Saccharin exposure resulted in glucose intolerance through considerable dysbiosis. Many increased taxa belonged to *Bacteroides* and *Clostridiales*, but *Firmicutes* and *Cyanobacteria* were decreased [30,66]. Similarly, metabolic change could not be induced in germ-free mice, while it occurred after transplantation with gut microbiota. Saccharin would also result in liver inflammation, causing increases in *Akkermansia*, *Corynebacterium* and *Turicibacter* and decreases in *Anaerostipes*, *Ruminococcus* and *Dorea* [67]. Moreover, the combined use of saccharin, glyphosate and sodium benzoate caused increases in the number of *E*. *coli* and *Pseudomonas* genera [97]. There was an increase in the population of the *Lactobacillaceae* and a reduction in the *Ruminococcaceae* family after exposure to SUCRAM (saccharin + neohesperidin dihydrochalcone) [98,99]. Similar results were obtained in vitro [65]. Additionally, the growth of *Faecalibacterium prausnitzii* and the *E*. *coli* strains *HB101* and *K-12* were also reduced [26,92].

Saccharin supplementation might significantly inhibit the growth of gut bacteria and improve dextran sulfate sodium (DSS)-induced colitis (Table 1). Exposure to saccharin influenced β-diversity and microbiota compositions showed a higher level of *Bacteroidetes* and a lower level of the *Firmicutes* phylum. The increase in anti-inflammatory bacteria *Bacteroides* and *Parasutterella* genera were observed. The research also discovered the bacteriostatic effect of saccharin on the growth of *Staphylococcus aureus*, *Klebsiella pneumonia* and *Pseudomonas aeruginosa* in vitro [30]; saccharin would affect the metabolomic profiles in feces (Table 2). The contents of daidzein, dihydrodaidzein and odesmethylangolensin were elevated, while equol was reduced in feces. Compounds that might mediate inflammation such as linoleoyl ethanolamide, palmitoleoyl ethanolamide, N, *N*-Dimethylsphingosine and quinolinic acid were strikingly altered [67]. Moreover, levels of propionate, acetate as well as phenyllactic acid were markedly higher, but the formic and benzoic acid were reduced [65,66]. The functional enrichment analysis indicated that LPS biosynthesis, flagellar assembly, fimbrial synthesis, bacterial toxin and multidrug resistance were possibly relevant [66]. Recently, NAS including saccharin, sucralose, aspartame and Ace-K were found to promote the bacterial evolution and horizontal transfer of antibiotic tolerance through natural transformation, resulting in an overexpression of genes encoding DNA uptake and translocation machinery [100,101,102]. This finding offers some insights into the roles of NAS in the evolution and dissemination of antibiotic tolerance among bacteria. Moreover, artificial sweeteners may inhibit the quorum sensing of the intestinal bacterial community, affecting the normal group behaviors [103]. According to our speculation, saccharin may interplay with gut microbiota and their metabolites, resulting in gut inflammation. 

### 2.5. Neotame (E 961)

Neotame tastes 7000–13,000 times sweeter than sugar. The intake of neotame decreased the α-diversity and changed the β-diversity of the fecal microbiome. An extreme decline in *Firmicutes* was observed. The abundance of *Bacteroidetes,* especially the *Bacteroides* genus, was enhanced. Notably, multiple components of the *Lachnospiraceae* and *Ruminococcaceae* families were significantly reduced, including *Blautia*, *Dorea*, *Oscillospira* and *Ruminococcus* genera. Neotame consumption also altered two butyrate fermentation pathways of gut microbiome. One of the pathways included the decreases in three genes encoding t4-hydroxybutyryl-CoA dehydratase, butyryl-CoA dehydrogenase and acetate CoA-transferase. These genes participated in the process of succinate fermentation to butyrate. For the other one, upstream genes regarding butyrate fermented from pyruvate were also downregulated. Additionally, the enriched pathways included amino acid metabolism, LPS biosynthesis and antibiotics biosynthesis, while fatty acid and carbohydrate metabolism pathways were reduced [68]. There were decreases in most lipids and fatty acids such as 1,3-dipalmitate, 1-monopalmitin, linoleic acid and stearic acid (Table 2). In feces, the lower content of cholesterol, campesterol and stigmastanol was found [68].

For our considerations, the enrichment of folate synthesis and LPS biosynthesis pathways is probably due to the increases in *Bacteroides* and *S24-27*. *Lachnospiraceae* and *Ruminococcaceae* were regarded as plant degrading and SCFAs-producing bacteria. The downregulated genes in butyrate synthesis and a lower amount of *Lachnospiraceae* and *Ruminococcaceae* possibly suggested a reduced production of SCFAs, thus declining lipid and fatty acid absorption. However, metabolic outcomes by neotame are still inadequately understood. Although the alteration of gut microbiota is similar to that in patients with IBD, the relation between neotame and intestinal inflammation remains unclear. Future studies are necessary to explore the effects of long-term exposure in colitis models or in humans.

### 2.6. Cyclamate (E 952)

Cyclamate is the sodium salt of cyclamic acid. It can be transformed to cyclohexylamine by the intestinal microbiota and eliminated from the feces. This food additive had been removed from the generally recognized as safe (GRAS) list since 1970, while it was considered safe by EFSA until now. A previous study revealed that the intake of cyclamate did not alter the compositions of fecal bacteria (e g. *Bacteroidaceae*, *Bifidobacteria*, *Lactobacilli*) [104]. However, in the in vitro model GIS1, there were increases in the *Bifidobacterium* and *Pediococcus* genera. A decrease in total SCFAs, especially the ratio between butyric and propionic acids, was also observed (Table 2) [65], while cyclamate inhibited the anaerobic fermentation of glucose [105]. As we know, SCFAs have beneficial influences on human health. Butyric acid may be effective against obesity and insulin resistance and can promote dyslipidemia. Propionic and butyric acids have been shown to be beneficial for IBD in a low concentration [27]. Overall, the conclusions on the total effect of cyclamate on gut microbiota and IBD cannot be drawn, and more studies are needed to figure out its impact on intestinal inflammation.

## 3. Sugar Alcohols

Sugar alcohols, or polyols, are low-calorie sweeteners that contain about half of the calories of white sugar and only lead to a slight alteration of blood sugar. Most of them are not well absorbed or metabolized in the host and are usually fermented by microbiota in the colon. Among them, erythritol, isomalt, xylitol and mannitol are commonly used as food additives.

### 3.1. Erythritol (E 968)

**Figure 1:**
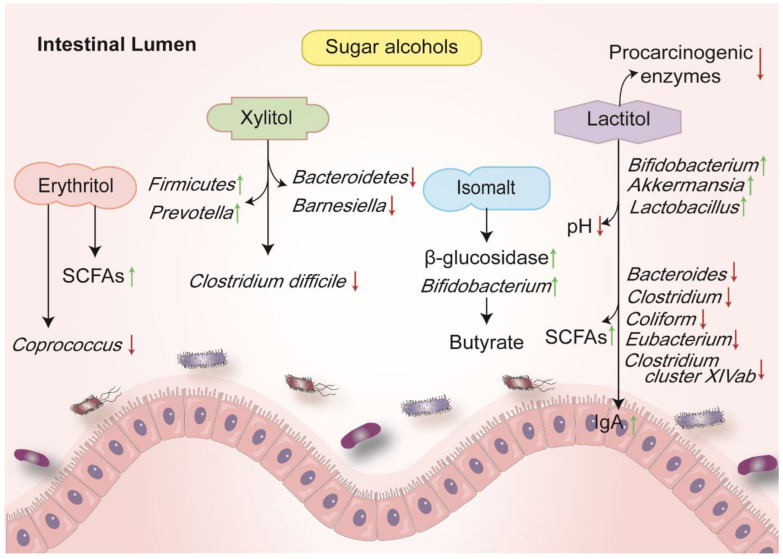
The impact of sugar alcohols on gut microbiomes and metabolites. “↑” means an increased level; “↓” means a decreased level.

Erythritol can be found naturally in fruits. After an intake of erythritol, about 90% can be absorbed into the small intestine with a very low metabolization and excreted unchanged through the urine. In the colon, intestinal flora can metabolize the unabsorbed part [106]. Human gut microbiota incubated with erythritol do not change the total gas production, pH values or SCFAs production [107]. Karley et al., demonstrated that the intake of erythritol caused increases in butyric and pentanoic acids with no significant changes of gut microbiota structure [69]. However, erythritol might ameliorate small intestinal inflammation in high-fat diet models, inducing a lower abundance of the *Coprococcus* genus [108,109]. The concentrations of SCFAs in the serum, feces and white adipose tissue were obviously elevated (Figure 1) [109]. Considering a limited amount of erythritol that reached the large intestine, the compound only slightly influences the compositions of gut microbiota. However, it may strengthen the gut microbiota to produce SCFAs, which might alleviate the intestinal inflammation. Although there is a lack of direct evidence about erythritol on IBD, it is considered a bacteria-friendly polyol to stabilize gut microenvironment and it can be degraded into IBD-friendly metabolites. 

### 3.2. Isomalt (E 953)

Isomalt has been used as a sweetener in the food and pharmaceutical industry for a long time. The microorganism can easily degrade the unabsorbed isomalt in the colon [110]. Recent studies considered it a prebiotic [70]. In humans, isomalt fermented in the gut promoted the abundance of *Bifidobacteria* and lowered the activities of bacterial β-glucosidase (Figure 1). SCFAs, lactate, bile acids, neutral sterols, N, NH_3_, phenol and *p*-cresol in feces were also altered (Table 2) [70]. The isomalt could be fermented by some *Bifidobacteria* strains in vitro, yielding a higher content of butyrate. However, no different gene expression was found after exposure to isomalt [70]. In another study, human intestinal microbiota was cultured with buckwheat honey, which is a crucial natural sweetener consisting of oligosaccharides and a small dose of isomalt and isomaltotriose. The study demonstrated that buckwheat honey could also increase the level of *Bifidobacteria* and restrain the pathogenic bacterium [111]. Although few clinical trials explore the effect of isomalt on IBD, isomalt may be considered as a bifidogenic polyol and a “close friend” for the intestinal homeostasis and microenvironment.

### 3.3. Xylitol (E 967)

Xylitol is a sugar alcohol with five carbons produced by the reduction of xylose. Oral microbial flora hardly get energy from xylitol, and it is considered as a noncariogenic sweetener and used to be applied to gum [112,113]. Xylitol was reported to affect the intestinal flora and have inhibitory effects on LPS-induced inflammatory cytokine expression. In a human study, the intake of the dietary low-digestible carbohydrates including xylitol led to a marked elevation of *Anaerostipes* spp. and butyrate in feces [114]. Xylitol consumption could shift rodent intestinal microbial population from Gram-negative to Gram-positive bacteria [115]. Xylitol lowered the level of fecal *Bacteroidetes* phylum and the *Barnesiella* genus, whereas increased the abundance of *Firmicutes* phylum and the *Prevotella* genus [114,116]. Lower xylitol concentrations could also inhibit the harmful mutans *Streptococci* (Figure 1) [117]. Hence, we suppose that xylitol may be beneficial for the growth and metabolism of intestinal flora without producing low harmful stimulations on intestine.

### 3.4. Lactitol (E 966)

Lactitol is regarded to be of mild sweetness and shows a lower sweetness than lactulose. Similar to xylitol, it is also considered noncariogenic and cannot be metabolized in the upper digestive tract for the deficiency of β-galactosidase; however, saccharolytic bacteria in the colon is capable of degrading it to acquire energy. Several studies have regarded lactitol as a prebiotic, while higher intakes of lactitol may cause laxative effects [71,118].

For a healthy human, the intake of lactitol decreased the population of proteolytic bacteria such as *Bacteroides*, *Clostridium*, *Coliforms* as well as *Eubacterium* and increased the growth of *Bifidobacterium*, *Lactobacillus* and *Streptococcus* [72]. A small dose of lactitol could play a beneficial role for fecal microorganisms [72,73]. The supplement of lactitol increased the level of *Bifidobacterium* (including three species: *B*. *longum, B*. *pseudocatenu* and *B*. *latum*) and *Lactobacillus* (including three species: *L*. *salivarius*, *L*. *fermentium* and *L*. *oris*) but decreased the abundance of *Klebsiella pneumonia* in patients with liver cirrhosis [119]. There showed an improved abundance of *Bifidobacterium* and the reductions of the *B*. *coccoides*–*E*. *rectale* group and *Clostridium cluster XIVab* after the consumption of lactitol with *Lactobacillus acidophilus NCFM*. These combined prebiotic products might also improve mucosal functions in the colon [120,121]. The prebiotic UG1601 (composed of inulin, lactitol and aloe vera gel) improved chronic constipation symptoms, enhanced the relative abundance of butyrate-producing bacteria including *Roseburia hominis* and reduced the abundances of the *Firmicutes* phylum and the *Lachnospiraceae* family [122]. Another study also showed its beneficial effect in elevating the level of *Bifidobacterium*. This suggested that lactitol may be a promising prebiotic candidate for patients with constipation [71]. Similar gut microbiota alterations were also found in patients with chronic viral hepatitis after lactitol exposure [123]. Details are depicted in Figure 1.

In animals, the consumption of lactitol exerted a prebiotic effect and reduced the *Enterobacteriaceae* population [74,75]. Notably, the intake of lactitol ameliorated colitis, altered the α diversity of the gut microbiota and induced higher levels of *Akkermansia* (Table 1) [31]. This was different from the alterations of intestinal floras in IBD. *Akkermansia* can degrade mucin, produce SCFAs and provide energy for the host. They would improve inflammatory response and insulin resistance, protecting intestinal epithelium and mucosal barrier in obese and diabetic patients. The genome of *Akkermansia* had shown its ability to encode a variety of secretory proteins such as sulfates, proteases and glycol-hydrolyzes. Hence, *Akkermansia* was supposed to metabolize lactitol and improve self-proliferation.

The consumption of lactitol also changes the fecal metabolites (Table 2). It significantly reduced the pH level in feces [72,73,75], which might be the result of the alteration of SCFAs [71]. It also increased the IgA secretion without signs of mucosal inflammation [74]. Some carcinogenic enzymes such as azoreductase, 7a-dehydroxylase, β-glucuronidase, nitroreductase and urease were decreased. We suggested that this might be because of the reduction of anaerobic bacteria producing these enzymes such as *Bacteroides* and *Clostridium* and the replacement of *Bifidobacterium* or *Lactobacillus* [72]. 

All these results indicated that lactitol is applicable and noncariogenic as a prebiotic to improve gut dysbiosis and ameliorate intestinal inflammation, suggesting its acceptability for patients with IBD. 

## 4. Coating and Thickening Agents

Maltodextrin (MDX), a common coating and thickening agent in the food industry, is able to modulate microbial structure and host antibacterial defenses via multiple mechanisms. It was reported to significantly alter the diversity and abundance of gut microbiota [124]. In healthy individuals, the intake of MDX significantly increased the abundance of *Bifidobacterium longum* and *Bifidobacterium* spp. [125]. In a recent study, IL10 KO mice were pre-conditioned with fecal material from nucleotide-binding oligomerization domain 2 deficient (NOD2 KO) mice to elicit gut inflammation. After administration with 1%MDX, the fecal microbiome analysis indicated a significant shift in α-diversity and β-diversity. The goblet cells in the intestine of the MDX-fed mice had reduced mucin granule content, demonstrating the destruction of the mucosal barrier [32]. Animal exposure with MDX had higher incidences of necrotizing enterocolitis with mucosal villi erosions and inflammation; bacterial diversity was reduced, while there was a higher abundance of *Pseudomonas* spp., *Streptococcus* spp. and *Leuconostoc* spp. and a lower level of *Weissella app* after treatment [33]. More information is shown in Table 3. 

MDX is also capable of altering the proliferation and function of a specific kind of bacteria. In vitro, it might accelerate the biofilm formation of CD-associated adherent invasive *E*. *coli* (AIEC), which is uniquely presented in the mucosa-associated bacteria from patients with CD. MDX exposure induced type I pili expression and increased bacterial adhesion to human intestinal epithelial cell monolayers in a mechanism dependent on type 1 pili. The study also demonstrated an increased prevalence of malX, a gene essential for MDX metabolism in AIEC strains. This suggested that MDX metabolism might help colonization of *E. coli* in the terminal ileum [33]. Similarly, MDX wrecked the intestinal antimicrobial barrier and enhanced mucosal *Salmonella* colonization in mice with *Salmonella*-infected gastroenteritis. The study also found the inhibition of energy metabolism in murine bone marrow derived macrophages after the exposure of MDX with the downregulated expression of NAPDH oxidase and limited recruitment of NADPH oxidase to *Salmonella*-containing vesicles. It would also result in the persistence of *Salmonella* in enlarged Rab7+ late endosomal vesicles, which might explain the discovery in vivo [34]. However, another study showed that MDX promoted endoplasmic Q2 reticulum stress–driven mucus depletion and exacerbated intestinal inflammation without significant changes in mucosa-associated microbiota (Table 1) [126]. 

Different patterns of MDX also exert distinct influences on intestinal flora. Longer MDX chains were more effective for the biofilm formation of AIEC, while short-clustered MDX attenuated metabolic dysregulation, causing a possible probiotic effect [35]. Isomaltodextrin, enzymatically produced from MDX by the enzymes α-glucosidase and α-amylase, promoted the growth of *Bifidobacteria* in the cecum and was supposed to be anti-inflammatory [127]. MDX also altered the concentration of SCFAs, although there is a lack of conclusive evidence about how MDX impacted bacterial metabolites (Table 2) [17,33,76,128]. In total, MDX could trigger intestinal inflammation with the proliferation and colonization of some harmful genera, while promoting the growth of some beneficial microbiota. However, recent evidence mainly originated from animal experiments and more studies are needed to clarify its effect in humans.

## 5. Emulsifiers

Emulsifiers are commonly applied in our daily life for their ability to stabilize emulsions and prolong shelf life by preventing separation during storage. They can act as gelling agents and surfactants via the fat molecules in food, adsorbing to the hydrophobic end of the emulsifiers and water adsorbing to the hydrophilic end. Common emulsifiers include carboxymethylcellulose (CMC), polysorbates, carrageenan, etc., [129,130,131].

### 5.1. Carboxymethylcellulose (E 466) and Polysorbate 80 (E 433)

CMC and polysorbate 80 (P80) are commonly found in edible oils, ice cream, cake mixes, icing and chocolate syrup. Nevertheless, these additives do not deserve a seat for intestinal homeostasis. Exposure to emulsifiers CMC and P80 negatively impact intestinal microbiota [131].

The emulsifiers may drive gut inflammation through microbiota (Table 1). P80 administration caused similar alterations to human gut microbiomes as IBD, resulting in a reduction of the beneficial *Bifidobacterium* genus, important SCFAs producers such as *Faecalibacterium* and *Subdoligranulum* genera, as well as *Clostridium leptum* [17]. Naimi et al., treated the fecal samples from a healthy participant with different emulsifiers in the MiniBioReactor Array model ex vivo, and they found the altered bacterial β-diversity after treatment with P80, with the lower growth of *Streptococcus* and *Faecalibacterium* [124]. In mice, an intake of P80 exacerbated indomethacin-induced ileitis, decreasing the α-diversity of intestinal microbiota. The growth of the sulphide producers including *Enterobacteriacaeae* and the swarming behavior of IBD-related species *Proteus mirabilis* were significantly promoted [36]. Swarming is a type of flagella-mediated movement that is a multicellular process and requires the differentiation of vegetative cells into a specialized cell type called a swarmer cell [132]. The swarming ability of *Proteus mirabilis* is highly related to its pathogenesis in IBD [133]. Interestingly, antibiotic pretreatment abolished this harmful effect, suggesting the important role of ileal dysbiosis [25]. Human microbiota meta-transcriptome after P80 exposure showed that biological processes including nucleic acid binding, structural constituent of ribosome, ion binding, nucleotide binding, isomerase and oxidoreductase activity were enriched [124]. 

A recent clinical trial investigating the effect of CMC on gut microbiota in humans reported that this exposure altered the bacterial richness and diversity, leading to an elevated level of *Roseburia* spp. and *Lachnospiraceae* as well as a lower amount of *Faecalibacterium prausnitzii* and *Ruminococcus* spp. [134]. In the study from Naimi et al., the β-diversity of human gut microbiota was significantly changed with the decrease in *Streptococcus* after CMC treatment [124]. The addition of CMC also induced severe CD-like colitis in IL10 KO mice. CMC changed the β-diversity of gut microbiota and enhanced the concentrations of total bacteria in the ileum. Moreover, there were more bacteria filling the gap between villi, increasing their migration to the bottom of the crypts [11,38]. Chassaing et al., discovered that the protracted intake of CMC or P80 induced low-grade intestinal inflammation and promoted robust colitis in mice predisposed to this disorder, ultimately resulting in the increased bacterial encroachment. These emulsifiers induced a reduction in microbial diversity, upregulating the richness of *Akkermansia muciniphila* and the inflammation-promoting bacteria *Proteobacteria*. After transplanting with the fecal materials from emulsifier-treated animals, germ-free mice showed a moderate inflammation in the colon with the alteration of gut microorganisms. This further illustrated the role of emulsifiers in promoting dysbiosis-driven pathologies [11]. The authors further applied for the mucosal simulator of the human intestinal microbial ecosystem model (M-SHIME) to investigate the alterations of human microbiota composition and gene expression ex vivo. The results showed that P80 drastically influenced microbiota diversity and composition, while CMC exhibited a clear effect on the composition of a complex microbiota. Moreover, CMC-treated and P80-treated M-SHIME suspensions were both capable of promoting low-grade gut inflammation in germ-free mice, promoting the growth of inflammation-associated bacteria such as *Proteobacteria* and *Enterobacteriacae* and reducing the levels of *Bacteroidaceae* (Table 1) [39]. 

**Figure 2:**
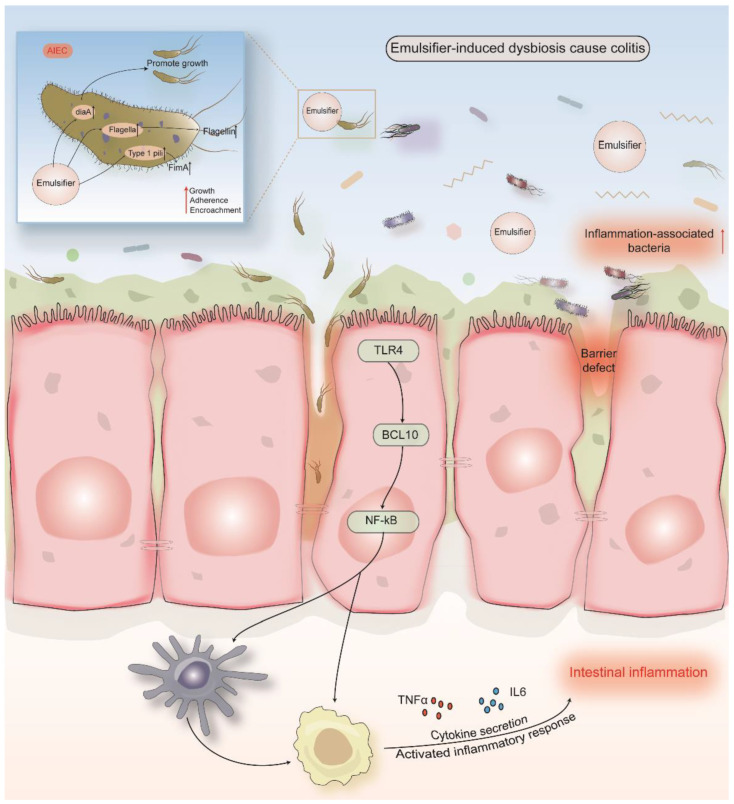
Mechanism of emulsifier-induced colitis via gut microbiome. Emulsifiers (1) altered the growth and functions of AIEC, resulting in the activation of inflammatory pathways in the epithelium; (2) enhanced the abundance of inflammation-related bacteria; (3) destroyed the mucosal barrier. “↑” means an increased level.

Many studies also investigated the interaction between emulsifiers and *E coli*. AIEC alone is sufficient to make mice prone to detrimental impacts of CMC and P80. After consuming CMC and P80, AIEC colonization elicited chronic intestinal inflammation and intestinal bacterial encroachment in germ-free mice [10]. Exposure to emulsifiers also increased its adherence to intestinal epithelial cells in vitro. Such effects are more pronounced when additional microbiota, such as altered Schaedler flora members, exist [10]. Moreover, transcriptomic analysis revealed the upregulation of genes that mediate the AIEC virulence and its ability to promote inflammation. Both emulsifiers were able to induce the expression of diaA, which can encode a DnaA initiator-associated protein. Genes related to flagella, type 1 pili and long polar fimbriae were notably upregulated by CMC in a dose-dependent manner [10]. Moreover, the intake of CMC decreased porcine mucus pore size, leading to the slower particle diffusion rates through mucus. Nevertheless, P80 seems minimal in impacting mucosal microstructure and particle dispersion [135]. P80 increased the motility of *E coli* and its ability to translocate across microfold epithelial cells, through which the gut epithelium was invaded by intestinal floras [37]. To conclude, CMC and P80 interacted with pathogenic bacteria, promoting its virulence and encroachment. The potential mechanism is demonstrated in Figure 2. The metabolites in feces or luminal content were also changed after the intake of CMC and P80. Most SCFAs such as butyric, propionic, valeric and caprylic acid were obviously increased [11,17]. Fecal bile acid composition was drastically altered with the exposure to CMC [11]. The levels of flagellin and LPS were enhanced after treatment with both emulsifiers, which conformed to the metagenomes results showing an enrichment of genes related to flagella and bacterial motility in gut microbiome (Table 2) [10,32].

For patients with IBD, the intake of CMC and P80 should be thoroughly concerning due to its ability to destroy the mucosal barrier and to promote robust colitis via altering the compositions and functions of gut bacteria. More evaluations should be conducted to estimate their effects on gut health in human.

### 5.2. Carrageenan (E 407)

Carrageenans are a group of sulfated polygalactans which are GRAS for routine use [136]. They are commonly found in flavored milks, iced coffee, dairy-based ice cream and frozen desserts [137]. The re-evaluation of carrageenan by EFSA demonstrated that the current ADI of carrageenan should be considered temporary, and the opinion needs to be improved within 5 years. The metabolism of carrageenan is largely performed by gut microbiota in the host [138]. Hence, gut microbiomes directly interact with carrageenan, influencing the intestinal homeostasis. Interestingly, different kinds and molecular weights of carrageenan have various effects on the host. Carrageenan can be divided into low or high molecular weight, degraded- or undegraded-carrageenan [139,140]. Low molecular weight carrageenan has been shown to increase intestinal permeability and to be associated with the onset of colitis [40]. However, high molecular weight carrageenan might have promising antitumor and antioxidant activities. Carrageenan was reported to induce and aggravate intestinal inflammation, altering gut microbiota compositions (Table 1). Onderdonk et al., showed that carrageenan induced cecal ulcerations in piglets unless in a germ-free state [46,47]. The intake of carrageenan resulted in increases in *Proteobacteria* and *Deferribacteres*, as well as decreases in *Firmicutes*, *Actinobacteria*, and *Bacteroidetes* phylum [45]. Different isomers of carrageenan (κ-, ι- and λ-) were all suggested to harmfully impact gut ecology. For human gut microbiota, every isomer induced the changes of α-diversity and increased the microbiota proinflammatory potential. *Bacteroides* was significantly enriched by κ- and λ-carrageenan, while *Faecalibacterium* was decreased by ι-carrageenan, with a higher content of flagellin after treatment [124]. κ-carrageenan induced robust colitis in the high-fat diet model, significantly increasing *Shigella* and decreasing *Bifidobacterium* [17]. The abundance of two inflammatory-related bacteria *Alistipes finegoldii* and *Bacteroides acidifaciens* were remarkedly enhanced as well [42]. Likewise, rats fed with ι-carrageenan indicated a significant reduction of total bacterial abundance and the concentrations of *Enterobacteria* spp., *Staphylococci* spp., *Streptococci* spp. and *Lactobacillus* spp. in feces [141]. Shang et al., directly compared the influence of different isomers on intestinal floras. All of them remarkably induced a decrease in *Akkermansia muciniphila*. However, the richness and diversity of fecal bacteria was increased after ι-carrageenan treatment, but a reduction of both indices were observed after κ-carrageenan exposure [41,43].

Some studies draw an opposite conclusion. The addition of *Sarconema filiforme*, the red seaweed mainly containing ι-carrageenan, attenuated symptoms of metabolic syndrome and slightly modulated gut microbiota in rats [142]. In *Drosophila*, ι-carrageenan strikingly enhanced the abundance of *Commensalibacter*, which could downregulate NF-kB-dependent antimicrobial peptide genes and adjust commensal gut mutualism [143].

Moreover, previous studies supposed that the alterations of gut microbiome were only observed in carrageenan administered in drinking water but not in a carrageenan-supplemented diet, suggesting that binding to other food ingredients such as protein possibly changed its conformation and removed its bioavailability to some bacteria [42]. 

After immunizing the animals with *Bacteroides vulgatus,* the exposure of carrageenan led to more severe intestinal lesions, with the discovery of antibodies to these bacteria in the serum [47]. The activities of some enzymes such as azo reductase, β-glucosidase and nitro-reductase were decreased in the cecal contents [141]. In another study, two synergistic strains, *Bacteroides xylanisolvens 38F6A4* and *E*. *coli 38F6C1,* were obtained from the feces of a healthy person and administrated to germ-free mice, aggravating the gut inflammation when consumed with carrageenan. *Bacteroides xylanisolvens 38F6A4* could produce β-carrageenase for the degradation of carrageenan. Moreover, *E*. *coli 38F6C1* could indirectly contact with *Bacteroides xylanisolvens 38F6A4*. It would rapidly consume the oxygen during fermentation and provide a relatively hypoxic environment that favored the growth of *Bacteroides xylanisolvens 38F6A4*. These studies suggested that certain gut microbes might contain carrageenases that can interact together to degrade carrageenan and thus generate harmful metabolites, altering adaptive immune responses in the host [40]. The glycoprotein deleted in malignant brain tumors 1 (DMBT1) has been reported to interact with carrageenan. DMBT1 is a secreted glycoprotein displaying a broad bacterial-binding spectrum and helps prevent bacterial encroachment. The exposure of carrageenan competed for DMBT1-mediated bacterial aggregation via binding to the specific peptide that recognized bacteria and disrupted the mucosal protection provided by DMBT1. This showed a novel mechanism that carrageenan was able to wreck the DMBT1-provided innate mucosal immune function, which could then trigger the onset or perpetuation of an inflammatory response to intestinal bacteria or bacterial antigens [44].

For patients with UC in remission, a higher rate of relapse was performed after carrageenan-containing diet treatment [140]. With the direct harmful effect of carrageenan on IBD patients and gut microbiota, we supposed that carrageenan might have harmful effects on IBD patients as a result of the disorder of gut microbiome and intestinal homeostasis.

### 5.3. Glycerol Monolaurate

Glycerol monolaurate (GML) is a natural glycerol monoester of lauric acid and is approved as a safe emulsifier by the FDA. In vitro, GML inhibits the growth and pathogenicity of bacteria, fungi and enveloped viruses [144]. Hence, GML is considered an antimicrobial-emulsifier that is commonly used in the general public. 

Jiang et al., exerted a series of investigations on how GML influenced gut microbiota and affected systemic inflammation. The GML consumption would upregulate the circulating levels of proinflammatory cytokines. This significantly changed the β-diversity and composition of gut microbiota as a result of the increased abundance of *Bacteroides acidifaciens* and the reduced level of *Akkermansia muciniphila* and *Lupinus luteus* [145]. However, GML modulated the indigenous microbiota in a dose-dependent manner. A high dose of GML (1600 mg/kg) upregulated the expression of anti-inflammatory TGF-β1 and IL-22, increasing the relative abundances of healthy core microbiota such as *Clostridium XIVa*, *Oscillibacter* and *Parasutterella* [48]. Amounts of 400 and 800 mg/kg GML also improved the richness of anti-inflammatory *Barnesiella* in the context of a DSS challenge. GML demonstrated a beneficial effect for metabolic syndrome and obesity [48,49]. GML attenuated high-fat diet-induced circulating LPS load and alleviated insulin resistance, with increases in *Bacteroides uniformis*, *Bifidobacterium pseudolongum, Akkermansia* and *Lactobacillus*. These genera and species are remarkably relevant to the metabolic improvements by GML [50]. Table 1 shows the interactions between GML, gut microbiota and gut inflammation.

The protective effect of GML on colitis and the potential dysbiosis-related mechanism were also assessed [48,51]. GML pretreatment is superior to GML cotreatment for colitis. Pretreatment with GML increased the abundance of *Lactobacillus* and *Bifidobacterium* in feces, with a higher level of propionic acid and butyric acid. It led to a more rapid and a better remission of colitis, resulting in the reconstructed microbial communities with an enhancement of fecal SCFAs (Table 2) [31]. In summary, colitis remission induced by GML is associated with altered gut microbiota patterns, suggesting that it might be a friendly companion for IBD.

## 6. Food Colorants

The food colorant market is valued at USD 5 billion in 2020 and is estimated to grow to USD 6.8 billion by 2025, with a compound growth rate (CAGR) of 5.4% [146]. Food colorants such as azo food dyes and titanium dioxide nanoparticles (TiO_2_ NPs) are commonly applied in food industries to make food more appealing and to protect against other contaminants. 

### 6.1. Titanium Dioxide (E 171)

TiO_2_ NPs are commonly used engineered nanomaterials, which would be commonly found in foods, ink and sunscreen. Food grade TiO_2_ NPs are whitening agents, which have been widely applied in food products [147]. More than 40% of them are capable of being swallowed when chewing commercial gums [148]. Yet, according to the update from the European Food Safety Authority (ESFA), TiO_2_ NPs are no longer considered safe when used as a food additive due to their genotoxicity [149]. Exposure to TiO_2_ NPs might also lead to gut barrier dysfunction [150], resulting in moderate gut inflammation and exacerbated immunological response [58]. They also showed an antibacterial effect on probiotic or symbiotic bacterium and exerted negative effects on human beings [151].

In humans, the addition of TiO_2_ NPs resulted in a modest decrease in community density [78] and induced the reduction of the dominant *Bacteroides ovatus* and *Clostridium cocleatum* [79]. The enhancement of *Firmicutes* as well as the reduction of *Bacteroidetes* were reported in several animal studies [52,53,54,59]. The abundance of *Proteobacteria*, *Cyanobacteria* and *Actinobaceria* were also increased [152]. At the genus level, TiO_2_ NPs caused a diminishment in the beneficial bacteria, including *Barnesiella*, *Akkermansia* and *Bacteroides,* in a dose-dependent manner [54]. *Barnesiella* was reported to remove pathogenic bacterium, to eliminate the colonization of *Enterococcus* that are resistant to vancomycin and to restrain antibiotic-resistant bacteria from spreading [153]. It could improve anticancer compounds such as cyclophosphamide [154]. The reduction of *Barnesiella* was probably related to the pathogenesis of IBD. Moreover, they caused inflammatory infiltration and mitochondrial abnormalities in the colon, leading to an increase in the *Turicibacter* genus and *Lactobacillus_gasseri* and *Lactobacillus NK4A136_group* in feces [56]. Notably, *Lactobacillus* is considered a major biofilm producer [57]. TiO_2_ NPs may therefore combine to the bacteria, triggering the formation of biofilm [155]. In IL 10 KO mice, *Lactobacillus gasseri* might exert obvious anti-inflammatory effects, possibly via its ability to produce manganese superoxide dismutases [156]. Thus, the enhancement of *Lactobacillus gasseri* is supposed to be the adaptive response to the inflammatory and oxidative stress state led by TiO_2_ NPs [157]. Similar results were also demonstrated in *Drosophila* and *zebrafish* [151,158]. Details are revealed in Table 4.

TiO_2_ NPs might exacerbate the severity of colitis and even consequent colon carcinogenesis via intestinal microorganisms. Treatment with TiO_2_ aggravated high-fat diet-induced mucus layer disruption, while the depletion of intestinal floras would eliminate these effects. TiO_2_ NPs consumption also induced the increase in *Firmicutes* as well as the reduction in *Lactobacillus* and *Bifidobacterium* genus in obese mice [52,53]. TiO_2_ NPs might worsen immune disorders by reducing the proportion of Tregs and CD4+ T cells [58]. Similarly, gut microbiota removal would rescue the gut inflammation induced by TiO_2_ NPs. In summary, TiO_2_ NPs are capable of triggering inflammation and changing the mucosal barrier via gut microbiomes, which might be related to different disease states including obesity and IBD (Table 1).

Different dosage or size of TiO_2_ NPs had various impacts on gut microbiota (Table 4). A lower dose of TiO_2_ NPs exposure shared some similar results with the studies on CMC and P80. There were significant increases in *Lactobacillus* and *Allobacullum* [56]. Some studies showed that a higher dose did not cause a significant change [59], while an acute high-dose TiO_2_ NPs exposure would elicit an inflammatory response in ileum and affect the level of mucosa-associated bacteria including *Lactobacillus* [55]. Moreover, a larger size (50 nm or 100 nm) of TiO_2_ NPs inhibited *Lactobacilli* growth more obviously than a smaller size (10 nm) [151]. The smaller size would induce a higher concentration of endocellular ROS, corresponding to the better antibacterial effect [56]. Different components of TiO_2_ NPs also had various effects on gut microbiota. The food grade TiO_2_ NPs had greater rutile structure, but the industrial grade TiO_2_ NPs were largely anatase. Gavage of rutile TiO_2_ NPs led to irregular villus epithelium cell arrangement with longer intestinal villi. It impacted the intestinal microbiota more profoundly than anatase NPs. At the genus level, rutile TiO_2_ NPs increased the level of *Rhodococcu*, and anatase TiO_2_ NPs enhanced the abundance of *Bacteroides* [159]. Moreover, food grade TiO_2_ NPs exposure had a greater inhibitory effect on the shift from *Proteobacteria* to *Firmicutes* phylum [77].

TiO_2_ NPs alter the metabolites in feces (Table 2). Although three studies indicated no significant difference in SCFAs levels [17,78,79], two studies showed a reduction in SCFAs concentrations in mice exposure with a lower dose of TiO_2_ NPs [19,57]. However, a high dose of TiO_2_ NPs increased the fecal SCFAs level [55]. Fecal metabolomic analysis showed the increases in caprolactam, *N*-acetylhistamine and glycerol-phosphocholine as well as the decreases in L-histidine, 4-methyl-5-thiazoleethanol and L-ornithine [56]. The pathway of aminoacyl-tRNA biosynthesis was remarkedly enriched in bioinformatics analysis. Aminoacyl-tRNAs are the essential substrates for translation in gut microbiota [66]. Moreover, it was demonstrated that TiO_2_ NPs alone could act as adjuvants, inducing cytokine responses through NLRP3 inflammasome activation and ROS generation when presented with bacterial antigens such as LPS [160].

In summary, the intake of TiO_2_ NPs will destroy the mucosal integrity and change the function and abundance of specific flora in the intestine, which is associated with the risk and relapse of IBD. 

### 6.2. Azo Dyes

**Figure 3:**
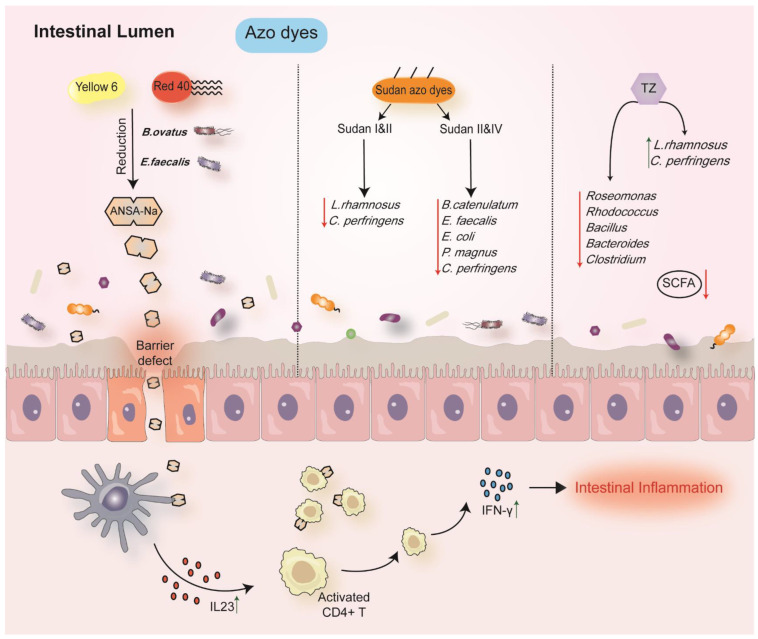
The influence of different food colorants on mucosal barrier and intestinal microecology. “↑” means an increased level; “↓” means a decreased level.

Azo dyes consist of a diazotized amine which is attached with a phenol or an amine and includes at least one azo linkage. In vivo, the azo bond of azo dyes can be cleaved by azo reductase to produce aromatic amines [161]. The food azo dyes include Allura Red (Red 40, numbered as E 129), Ponceau 4R (E124), Carmoisine (E 122), Quinoline Yellow (E 104), Tartrazine (TZ, numbered as E102), and Sunset Yellow (Yellow 6, numbered as E110) [162]. Red 40 was considered to exacerbate gut inflammation in colitis-prone mice [60], while the composition of fecal bacteria was not altered significantly (Table 1). However, they did not observe colitis in germ-free mice until colonized mice with *Bacteroides ovatus*, indicating that the proinflammatory effect of Red 40 depended on gut bacterium. Furthermore, Red 40 and Yellow 6 could be metabolized by commensal bacteria into 1-amino-2-naphthol-6-sulfonate sodium salt (ANSA-Na). Treatment with ANSA-NA also caused colitis in wild-type mice rather than germ-free mice. This suggested that the metabolite exerting the colitis-prone effect required the participation of bacteria (Table 2 and Figure 3) [60].

TZ consumption could cause severe physiological dysfunction and histopathological alterations in crucian carp. It might trigger the oxidative stress and upregulate the proinflammatory cytokines in a dose-dependent manner, indicating a tendency to intestinal inflammation. Moreover, a significant reduction was found in certain probiotic bacteria, including *Rhodococcus*, *Roseomonas* and *Bacillu*, while the pathogenic bacteria such as *Bdellovibrio* and *Shewanella* were enriched [61]. SCFAs, especially butyrate, also decreased after TZ uptake [80]. Hence, it is of significance to address the deleterious effect of TZ on intestinal barriers and microbiota, which might be hostile to patients with IBD (Figure 3).

Sudan azo dyes (Sudan I, II, III, IV and Para Red) have been commonly applied in making printing inks, plastics, leather, waxes and fabrics [82]. Due to their carcinogenicity, most countries have banned them from the food industry. They are still illegally used in some agencies due to their cheap price and bright coloring. Sudan I and II restrained the growing of *Lactobacillus rhamnosus* and *Clostridium perfringens*. Sudan II might influence the growth of *Enterococcus faecalis*; however, after being cultured with Sudan III and IV the content of *Bifidobacterium catenulatum*, *Clostridium perfringens*, *Enterococcus faecalis*, *Peptostreptococcus magnus* and *E*. *coli s* was significantly decreased in vitro. All of them are capable of selectively suppressing the survival capability of two *Clostridium* species (*Clostridium indolis* and *Clostridium ramosum*). Similar to TZ, one of the metabolites of the dyes, 1-Amino-2-naphthol, was able to inhibit the growing of most bacteria [82]. Sudan III and IV could be degraded into some carcinogenic aromatic amines such as aniline and o-toluidine by some common bacteria, respectively [81]. In summary, Sudan III and IV could inhibit the growth of human gut microbiota more strongly than Sudan I, II and Para Red. Current evidence show that Sudan azo dyes and their metabolites play hazardous roles on intestinal homeostasis. (Table 2 and Figure 3) [80,82]. 

## 7. Preservatives

Food preservatives can postpone degradation and prolong expiration time in foods, limiting the growth of microorganisms and inhibiting the food oxidation. Nowadays, many common preservations such as benzoic acid, potassium sorbate, sodium nitrite and sodium sulfite as well as Ag nanoparticles (Ag NPs) are also reported to induce the alteration of gut microbiota. However, the American Academy of Pediatrics cautions that preservatives may be associated with worsened hyperactive behavior or risk of carcinogenicity, suggesting some preservatives should be avoided in children. This finding has attracted the attention of the FDA in recent years [163,164,165,166,167].

### 7.1. Benzoic Acid and Sodium Benzoate (E210-213)

Both benzoic acid (BA) and sodium benzoate (SB) act as food preservatives due to their ability to limit the growth of pathogenic microorganisms. All the absorbed BA can be completely degraded into hippuric acid [168]. Recent studies have investigated the beneficial effect of BA and SB on intestinal barrier functions and gut microbiota by regulating oxidative status and the immune state. However, excessive intake might result in the destruction of the intestinal barrier through redox status. 

Treatment with BA enhanced the degree of biodiversity of ileal microbiota, lowering the abundance of total aerobic bacteria in a dose-dependent manner [83]. It would also reduce the richness of Gram-negative bacteria in the duodenum [84]. In piglets, treatment with BA increased the height of the intestinal villus, enhanced the level of beneficial microorganisms (e.g., *Bifidobacterium* and lactic acid bacteria) and decreased the population of harmful microorganisms [85,169]. The cecum content of piglets showed a similar effect, with lower concentrations of *Escherichia-Shigella* and a higher level of *Lactobacillus* and *Bacillus* [86,170]. However, a mixture of sorbate, benzoate and nitrite led to the thriving of *Proteobacteria* and the significant reduction in *Clostridiales*. Recent studies reported that BA increased the susceptibility to induce *Proteobacteria* dysbiosis in the NOD2 KO mice (Table 1). Remarkably, the proliferation of *Proteobacteria* is suggested as a potential diagnostic marker of dysbiosis and related to the risk of diseases such as IBD [62]. Moreover, hippuric acid was decreased in the urine, which found a reduction in patients with CD due to the alteration of gut microbiome (Table 2) [83]. 

The therapeutic potential of SB was observed in a UC model, possibly due to its antioxidant and anti-inflammatory activities. SB might selectively suppress the growth of susceptible gut microbes. The addition of benzoate and glyphosate in the diet might lead to the overgrowth of total intestinal microbiomes, especially the *Enterobacteriaceae* family [97]. Interestingly, *Enterococcus faecalis*, *Lactobacillus paracasei* and *Bifidobacterium longum* are more susceptible to SB [62,171]. We indicated that SB might be a friendly player in the gut microbiota of IBD. While the existing studies showed contradictory opinions on BB, more studies are needed to clarify their effects on intestinal flora and gut inflammation.

### 7.2. Potassium Sorbate (E 202)

Potassium sorbate (PS) is an antiseptic agent of low toxicity, strongly inhibiting spoilage bacteria and mold with slight effects on its organoleptic properties [172]. Previous studies have reported that PS restrained the viability of gut microbiota via the alteration in host immunity. Exposure to PS remarkably reduced the content of IgG, IL-1β and TNF-α in the gut, with the activation of the immune system in zebrafish. At the genus level, the content of beneficial bacteria *Faecalibacterium*, as well as pathogenic bacteria *Aeromonas* and *Methylobacterium,* demonstrated a significant downward trend [172]. In vitro, the growth of *Faecalibacterium prausnitzi* also significantly decreased after treatment with PS [92]. Moreover, most of the susceptible bacteria are Gram-negative after PS exposure. Notably, the lipophicity of PS plays an important role in modulating different types of gut microbiota. There is a large amount of lipid content in Gram-negative bacterial cell walls, thus inhibiting the pathway in transcription and carbohydrate metabolism. However, the Gram-positive bacterium contains a higher peptidoglycan content, possibly blocking the delivery of PS [172]. *E*. *coli* has been shown to be resistant to PS by its efflux pump mechanism [171]. The combination of benzoate, nitrite and sorbate significantly affected the susceptibility of intestinal microbes to preservatives in human, which suggested the pathogenesis of metabolic or immune-mediated diseases such as IBD [62,171]. However, more studies are needed to investigate the interplay between potassium sorbate, gut microbiota and IBD.

### 7.3. Sulfites (E 211)

Sulfites refer to metabisulfites including: hydrogen sulfites, sulfur dioxide gas and sulfur salts containing potassium, calcium or sodium [173]. Sodium sulfite (SS) is one of the most common food preservatives among them. It strongly inhibited the growth of *Faecalibacterium prausnitzii* [92]. *Lactobacillus casei*, *Lactobacillus rhamnosus* and *Lactobacillus plantarum*, considered as beneficial gut bacteria for their ability to produce lactic acid, were also reduced [173]. In another study, SS consistently decreased the viability of *Proteus mirabilis*, *Escherichia fergusonii*, *Morganella morganii* and *Klebsiella pneumoniae* obtained from patients with CD (Table 1) [63]. In summary, sulfites, especially SS, might have a stronger antimicrobial ability on beneficial bacteria in the gut, which may induce the intestinal disorders of IBD.

### 7.4. Ag NPs (E 174)

Since 1891, Ag NPs have been used as a wound antiseptic [174]. The mechanism for its antibacterial properties is believed to be that Ag ions interact with the thiol group of some essential enzymes of bacteria, suppressing the normal activities and physiological functions [175]. In vitro, Ag NPs showed a negative impact on the human bacterial community with a significant reduction in culture-generated gas production. Moreover, the abundance of *Ruminococcus torques*, *Roseburia intestinalis*, *Eubacterium rectale*, *Roseburia faecalis* and *Bacteroides ovatus* were remarkedly declined [176]. Another study found a decrease in *Enterococcus* spp. and lactic acid producing bacteria, as well as an increasement in *Clostridium* spp. after treatment with solid Ag NPs, while limited changes of bacterial composition and metabolism were found in an in vitro dynamic model [87].

Moreover, the α-diversity and β-diversity were disturbed in a dose-dependent manner, with the elevated ratio between *Firmicutes* and *Bacteroidetes* phyla. *Lachnospiraceae* and the *S24-7* family mainly accounted for the increase in *Firmicutes* and decrease in *Bacteroidetes*, respectively [177]. In another study, Ag NPs and silver acetate induced the reduction of the *Firmicutes* phylum and the *Lactobacillus* genus. Exposure to Ag NPs in a smaller size or at a lower dose might downregulate some immunomodulatory genes [178]. Interestingly, different shapes of Ag NPs such as cube shapes (AgNC) and sphere shapes (AgNS) might lead to varied effects. *Bacteroides uniformis* and *Clostridium* spp. were declined after AgNC exposure, whereas *Dehalobacterium* spp., *Peptococcaeceae* and *Oscillospira* spp. were altered in the AgNS-treated group [179]. However, a varied-coating dose did not significantly change gut microbiomes [180]. The bacterial alteration might be gender-specific in zebrafish according to a previous study [181]. 

Ag NPs limited the growth of the probiotic *Lactobacilli* and some common opportunistic bacteria such as *Staphylococcus aureus* and *E*. *coli*. *Lactobacilli* are more susceptible than the opportunistic bacteria when Ag NPs presented. *Lactobacilli* can provide an acidic environment, which promotes Ag NPs dissolution and can overproduce hydroxyl radical (•OH), eliminating the intracellular glutathione pool and elevating ROS levels. Higher ROS content will damage DNA and cause apoptosis. This study summarized the possible mechanism of a pH-Ag^+^-•OH bactericidal pathway, showing a new insight to explain the effect of Ag NPs on human health [182]. It seemed that Ag NPs exerted a more inhibitory influence on some beneficial or common bacteria and damage to the intestinal homeostasis. It is of great concern for IBD onset and flare-ups if consumed in a higher level.

### 7.5. Other Preservatives

ε-polylysine is composed of isopeptide linked with 25–40 L-lysine residues between ε-amino and α-carboxyl groups. Although it is not permitted as a food additive in most countries, the FDA has granted the status of GRAS to this product. A recent study discovered an alteration of the intestinal microorganisms, increasing the abundance of *Bacteroides*, *Oscillospira* and *Coprococcus*, as well as decreasing the level of *Ruminococcus* and *Lactobacillu* [183]. Triclosan (TCS) is also applied as an antimicrobial ingredient used in toothpaste, cosmetics, kitchenware and toys, but was banned in 2013 by the FDA. TCS was found to be associated with colon inflammation and tumorigenesis through altering the intestinal microflora and toll-like receptor 4 signaling pathway. TCS significantly lowers the α- and β-diversity (Table 1). At the phylum level, there is a declination in *Bacteroidetes*, *Actinomycetes* and *Cyanobacteria*, with an enhancement in *Firmicutes*. In addition, *Bifidobacterium* and *Bacteroides* were significantly decreased at the genus level after treatment [64].

## 8. Antioxidants

Rosemary extract (RE, numbered as E 392), extracted from Rosmarinus officinalis Linn, has multiple biological and pharmacological functions and can be used as antioxidants in food additives and medicines. RE has shown protective effects against oxidation, inflammation and microbiota imbalance [184]. Dietary supplements with RE elevated the digestibility of nutrients, improving antioxidant status and intestinal morphology in pigs. In cecum, an increased number of *Bifidobacterium* and *Bacteroidetes* and a decreased abundance of *E*. *coli* was shown [185]. Both lean and obese female rats demonstrated similar effects, with increases in the *Bacteroides*/*Prevotella* groups and in *Blautia coccoides*. Moreover, *Clostridium leptum* and *Bifidobacterium* were significantly altered in the lean rats. The fecal SCFAs increased in obese rats, while they decreased in lean rats [88]. RE also exerts beneficial impacts on diabetes and depression [186,187]. Rosmarinic acid and carnosic acid are two rosemary components. They showed an antiglycative and antioxidative effect in diabetic rats. RE not only prevented against inflammation and tissue damage, but also exerted prebiotic effects on gut microbiota, accompanied with the overgrowth of diabetes-resistant bacteria such as *Actinobacteria*, *Bacteroides*, *Faecalibacterium*, *Lachnospiraceae* and *Prevotella*, as well as decreased amounts of diabetes-sensitive bacteria such as *Firmicutes* and *Ruminococcaceae*. Rosmarinic acid demonstrated more effectiveness in relieving the metabolic symptoms than carnosic acid [186]. Moreover, RE enhanced the sequence proportion of *Lactobacillus* and *Firmicutes*, and reduced the sequence proportion of *Bacteroidetes* and *Proteobacteria* in feces from chronic restraint stress mice [187]. In accordance with the evidence above, rosemary extract did play a role in improving inflammatory status and modulating intestinal microbiota, which might be a confidence for patients with IBD. 

## 9. Conclusions

A growing number of studies investigate the interactions between gut microbiota and food additives, indicating that these interplays might be involved in the pathogenesis of IBD. After summarizing the collected information from the current studies, our review concluded that food additives exert multiple effects on gut microbiota and intestinal homeostasis, which may be associated with the onset and progression of IBD. Furthermore, food additives also remarkably mediated the alterations of bacterial functions. Taken together, sweeteners such as Ace-K, sucralose and saccharin might induce or exacerbate colitis via elevating the bacterial inflammatory potentials. Emulsifiers such as CMC and P80 were considered deleterious to intestinal health by altering the diversity of gut microbiota and increasing bacterial encroachment. The safety of food colorants should be re-estimated due to their negative effect of triggering intestinal disorder and dysbiosis; However, polyols and antioxidants included in this study seem beneficial for gut microbiota by improving the intestinal microorganism structures and functions. Aspartame may also be intestine-friendly for IBD, with an elevated level of probiotic bacteria. Although included studies showed different impacts of food additives on intestinal microbiomes and gut inflammation, it is not feasible to attribute the same results obtained in vitro and/or in animal models to humans. Hence, future research should replicate human physiological conditions based on bio-relevant models.

According to current evidence and regulations, unfavorable food additives such as food colorants and emulsifiers including CMC and P80 for patients with IBD must be cautiously considered, and polyols such as lactitol are acceptable. However, the influence of sweeteners and antioxidants on gut microbiota and colitis is uncertain in humans and further studies are urgently needed to validate. In addition, the issues of MDX and preservatives on health and intestinal floras should be highlighted, and more trials are recommended to clarify their definite effect in humans. Meanwhile, substantial revision for the GRAS process is also recommended, especially in special groups (including children, pregnant and lactating women, etc.). Moreover, all food safety agencies should mutually leverage their professional knowledge and expertise evaluations to access the missing information and recognize the knowledge gap of additive food. In conclusion, more studies are needed to elucidate the relationship between food additives, gut microbiota and IBD, and to understand the risks of IBD to which future generations are potentially exposed due to the consumption habits that current generations have.

## Figures and Tables

**Table 3:** The impact of maltodextrin on gut microbiota.

Reference	[17]	[124]	[32]	[33]	[34]	[76]
α-diversity						
Richness	-	↑	↓	-	-	-
Diversity	-	-	↓	-	-	-
β-diversity	-	S	S	-	-	-
Genus						
*Bifidobacterium*	↑					↑
*Bacteroides*						↓
*Mucispirillum*						↑
*Desulfovibrio*						↓
*Lactobacillus*						↑
*Enterococcus*						↓
*Faecalibacterium*						↑
*Akkermansia*						↑
*Roseburia*		↑				
*Streptococcus*				↑		↓
*Alistipes*						↓
*Leuconostoc*				↑		
*Pseudomonas*				↑		
*Weissella*				↓		
*Oscillibacter*						
Species						
*Escherichia coli*					↑	
*Blautia coccoides*	↑					

Abbreviations: “↑”—higher α-diversity or bacteria are more abundant; “↓”—lower α-diversity or bacteria are less abundant; S—significant difference found in β-diversity.

**Table 4:** The impact of titanium dioxide nanoparticles on gut microbiota.

Reference	[17]	[58]	[151]	[78]	[79]	[52]	[53]	[54]	[59]	[152]	[56]	[57]	[155]	[55]	[159]	[77]	Total	
																	↑	↓
α-diversity																		
Richness	-	N	-	↓	-	-	-	↓	-	↓	N	N	↓	-	N	-	0	4
Diversity	-	N	-	N	-	-	-	↓	-	↓	N	N	-	-	N	-	0	2
β-diversity	S	-	-	N	-	-	-	S	S	S	N	N	S	-	N	-	S = 5	
Phylum																		
*Bacteroidetes*		↑				↓	↓	↓					↑			↑	3	3
*Verrucomicrobia*								↓					↑				1	1
*Firmicutes*						↑	↑	↑					↑	↑		↑	6	0
*Proteobacteria*															↑	↓	1	1
*Actinomycetes*		↑								↑							2	0
*Cyanobacteria*										↑							1	
*Deferribacteres*										↑							1	
Genus																		
*Bifidobacterium*		↓				↓											0	2
*Bacteroides*								↓							↑		1	1
*Parabacteroides*												↑					1	0
*Lactobacillu*		↓	↑			↓						↑					2	2
*Prevotella*															↓		0	1
*Turicibacter*											↑						1	0
*Akkermansia*								↓									0	1
*Adlercreutzia*												↓					0	1
*Barnesiella*								↓									0	1
*Rhodococcus*															↑		1	0
*Lawsonia*													↑				1	0
*Allobaculum*												↑					1	0
*Enterobacteria*			↑														1	0
*Acetobacteria*			↑														1	0
Species																		
*Clostridium leptum*	↓																0	1
*Clostridium cocleatum*					↑												1	0
*Bacteroides ovatus*					↓												0	1

Abbreviations: “↑”—higher α-diversity or bacteria are more abundant; “↓”—Lower α-diversity or bacteria are less abundant; S—significant difference found in β-diversity.

## Supplementary Materials

The following supporting information can be downloaded at: https://www.mdpi.com/article/10.3390/nu14153049/s1, Supplementary Table S1: The impact of different artificial sweeteners on gut microbiota.

## Statement of Significance

To date, this is the first systematic review to narrate the potential molecular mechanisms of food additives–associated microbiota changes involved in inflammatory bowel disease (IBD). These results implied high risks and rapid progression of IBD in current and future generations due to the undesirable consumption habits.
