# Supplementary material for: Food Additives Associated with Gut Microbiota Alterations in Inflammatory Bowel Disease: Friends or Enemies?
Source: Nutrients. 2022 Jul 25;14(15):3049. doi: 10.3390/nu14153049 (PMC9330785; doi:10.3390/nu14153049)
Supplement: Supplementary file 1 [file nutrients-14-03049-s001.zip › nutrients-1800091-supplementary.pdf]

Supplementary Table S1. The impact of different artificial sweeteners on gut microbiota.

**Artificial Sweeteners**	**Aspartame**				**Acesulfame K**						**Sucralose**										**Saccharin**								
**Reference**	(15)	(17)	(18)	(19)	(15)	(22)	(23)	(24)	(25)	(26)	(8)	(17)	(22)	(24)	(26)	(28)	(30)	(31)	(32)	(35)	(32)	(37)	(38)	(39)	(40)	(41)	(42)	(43)	(44)
**α-diversity**																													
Richness	N	-	-	N	N	-	-	↑	-	-	-	-	-	↑	-	-	-	-	-	-	-	-	-	N	-	-	-	-	-
Diversity	N	-	-	N	N	-	-	-	-	-	-	-	-	-	-	-	-	-	-	-	-	-	-	N	-	-	-	-	-
**β-diversity**	S	-	-	S	S	-	-	S	-	-	-	S	-	S	-	N	-	-	-	-	-	-	-	S	-	-	-	-	-
**Phylum**																													
Firmicutes			↑					↑		↑	↓			↑	↑				↓	↑	↑			↓	↓				
Bacteroidetes										↓	↑				↓									↑	↑				
Verrucomicrobia								↓						↓															
Proteobacteria											↑							↑		↓				↑					
Actinomycetes											↓									↑									
Cyanobacteria																									↓				
Tenericutes																									↓				
**Family**																													
Porphyromonadaceae				↑																									
Lachnospiraceae								↑						↑										↑					
Ruminococcaceae							↓	↑						↑															↓
Lactobacillaceae																													↑
Oxalobacteraceae							↓																						
Enterococcaceae																													
Clostridiaceae																	↑								↑				
Christensenellaceae																	↑												
Porphyromonadaceae																								↑					
Prevotellaceae																									↑				
Peptococcaceae																									↑				
Rikenellaceae																									↓				
Veillonellaceae																													↓
**Genus**																													
Bifidobacterium		↑								↑					↑				↓		↑								
Clostridium							↓			N																			
Bacteroides							↑																	↑	↑				
Parabacteroides																									↑				
Anaerostipes							↑																			↓			
Sutterella							↑																						
Lactobacillu							↓																					↑	
Mucispirillum							↑																						
Desulfovibrio									↓																				
Enterococcus			↓																										
Parasutterella			↓																					↑					
Thermoanaerobacter			↑														↑												
Clostridium cluster XIVa													↓																
Shigella												↑																	
Bilophila												↑																	

Turicibacter																	↑									↑			
Ruminococcus																	↑									↓			
Akkermansia																	↑									↑			
Roseburia																	↑									↑			
Staphylococcus																	↓								↑				
Alistipes																								↓					
Dehalobacterium																	↓												
Anaerostipes																	↓												
Streptococcus																	↓								↓				
Pediococcus																			↓										
Sporosarcina																										↑			
Jeotgalicoccus																										↑			
Oscillospira																										↑			
Corynebacterium																										↑			
Adlercreutzia																										↓			
Dorea																										↓			
Aerococcus																									↑				
Odoribacter																									↓				
Pseudomonas																											↑		
Bacteroides/Prevotella		↓																											
**Species**																													
Akkermansia muciniphila								↓						↓															
Clostridium leptum			↑	↑																									
Escherichia coli																											↑		
Blautia coccoides		↑	↑																										
Peptostreptococcus stomatis																				↑									
Clostridium symbiosum																				↑									
Peptostreptococcus anaerobius																				↑									

Abbreviations: "↑" = higher α-diversity or bacteria are more abundant; "↓" = Lower α-diversity or bacteria are less abundant; S, significant difference found in β-diversity.
